# Supplementary material for: Nucleolar translocation of human DNA topoisomerase II by ATP depletion and its disruption by the RNA polymerase I inhibitor BMH-21
Source: Sci Rep. 2021 Nov 2;11:21533. doi: 10.1038/s41598-021-00958-4 (PMC8563764; doi:10.1038/s41598-021-00958-4)
Supplement: Supplementary file 1 — Supplementary Information. [file 41598_2021_958_MOESM1_ESM.pdf]

Supplementary Information

Nucleolar translocation of human DNA topoisomerase II by ATP depletion and its disruption by the RNA polymerase I inhibitor BMH-21

Keiko Morotomi-Yano [1] and Ken-ichi Yano [1] *

[1] : Institute of Industrial Nanomaterials, Kumamoto University

* : Corresponding author (E-mail: yanoken@kumamoto-u.ac.jp)

Supplementary Figure 1

A

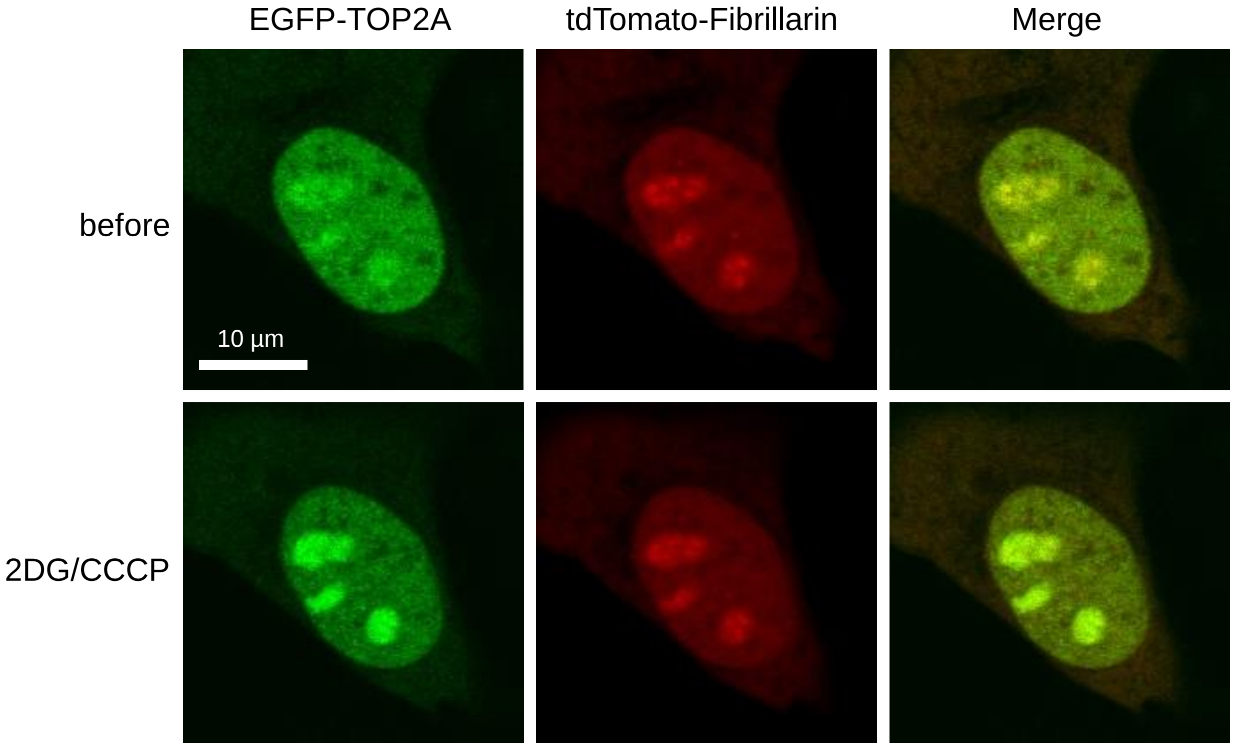

B

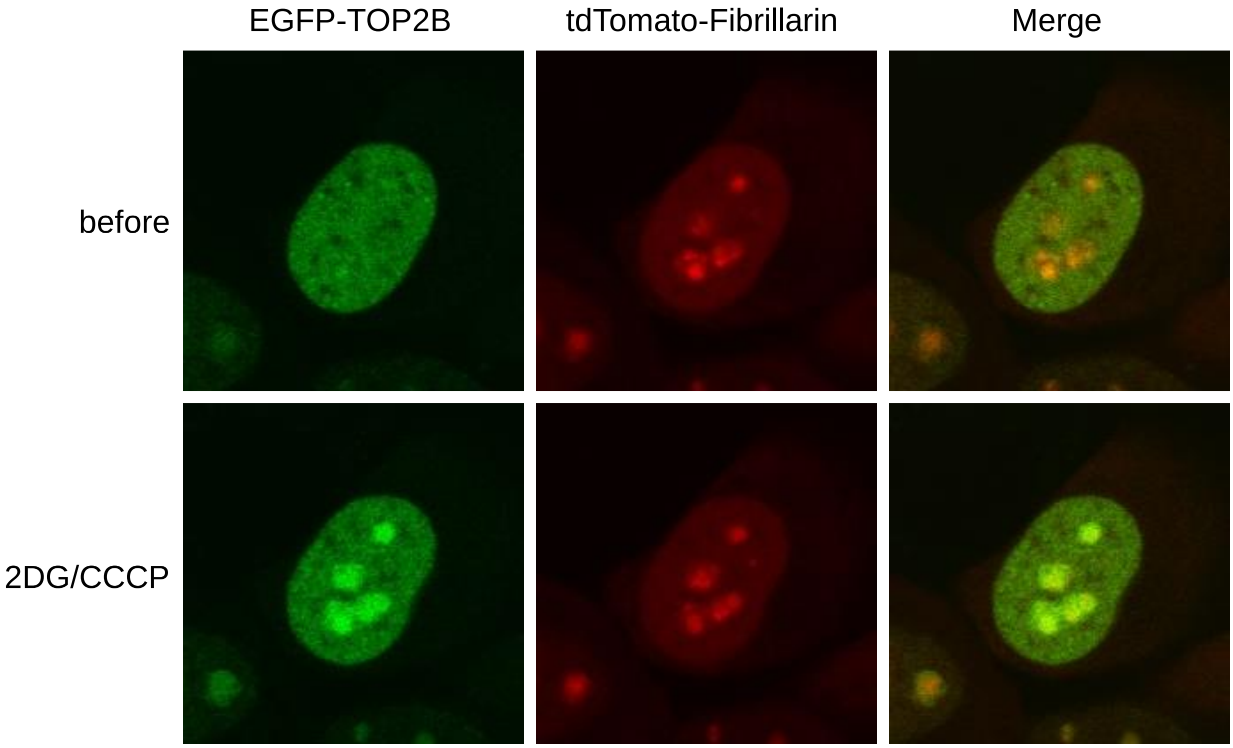

Supplementary Figure 1. Colocalization of EGFP-TOP2 proteins and tdTomato-Fibrillarin.

A. Expression plasmids for EGFP-TOP2A and tdTomato-Fibrillarin were cotransfected into HeLa cells. At 2 days after transfection, cells were treated with 25 mM 2DG and 10 μM CCCP for 20 min. Fluorescence images were captured as described in the main text, and representative images are shown. The tdTomato-Fibrillarin expression plasmid was obtained from Addgene (Plasmid #58093).

B. An experiment was repeated on EGFP-TOP2B and tdTomato-Fibrillarin as described above.

Supplementary Figure 2

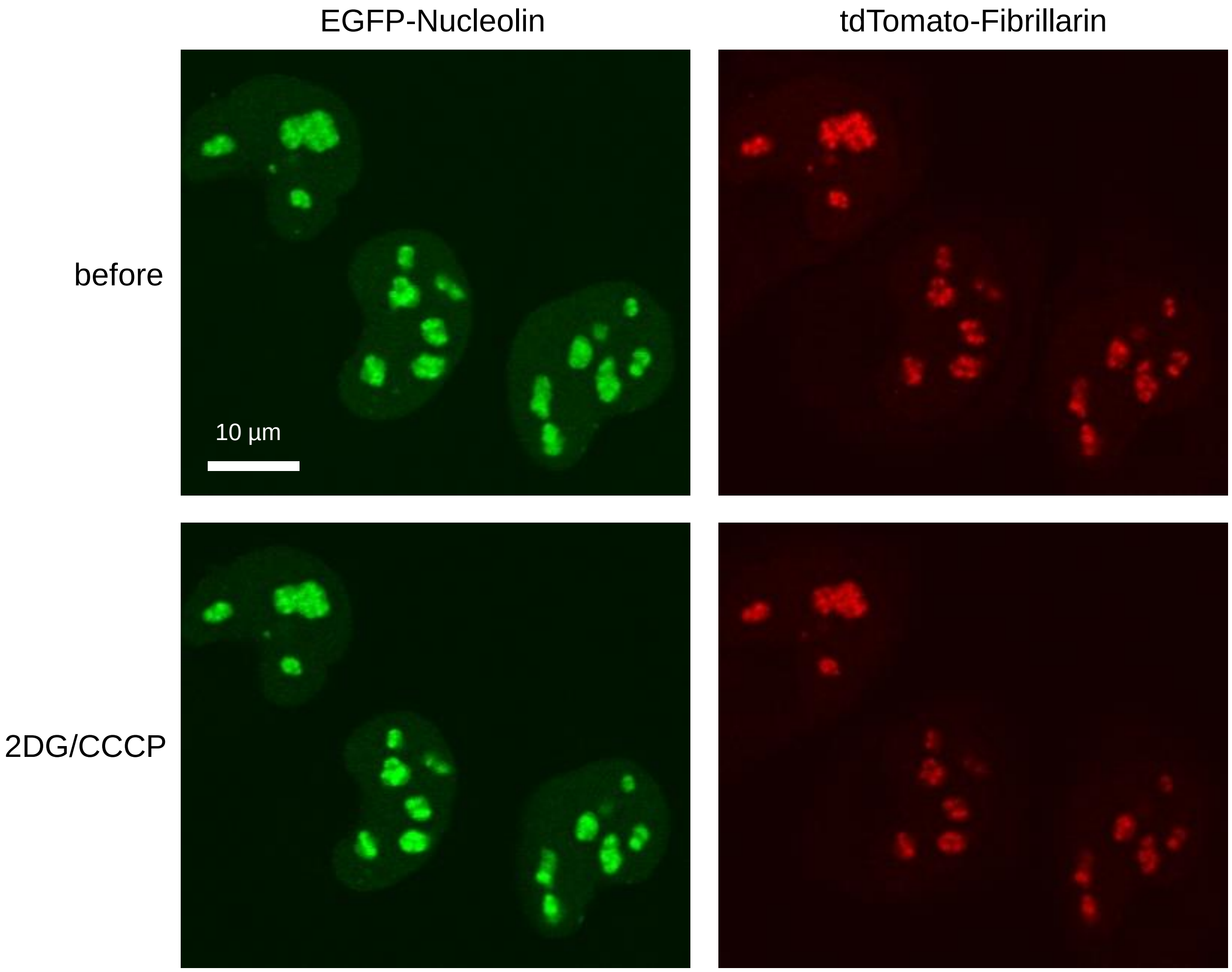

Supplementary Figure 2. 2DG/CCCP treatment does not affect the subnuclear localization of EGFP-Nucleolin and tdTomato-Fibrillarin.

Expression plasmids for EGFP-Nucleolin and tdTomato-fibrillarin were cotransfected into HeLa cells. At 2 days after transfection, cells were treated with 25 mM 2DG and 10 μM CCCP for 20 min. Fluorescence images were captured as described in the main text, and representative images are shown.

Supplementary Figure 3

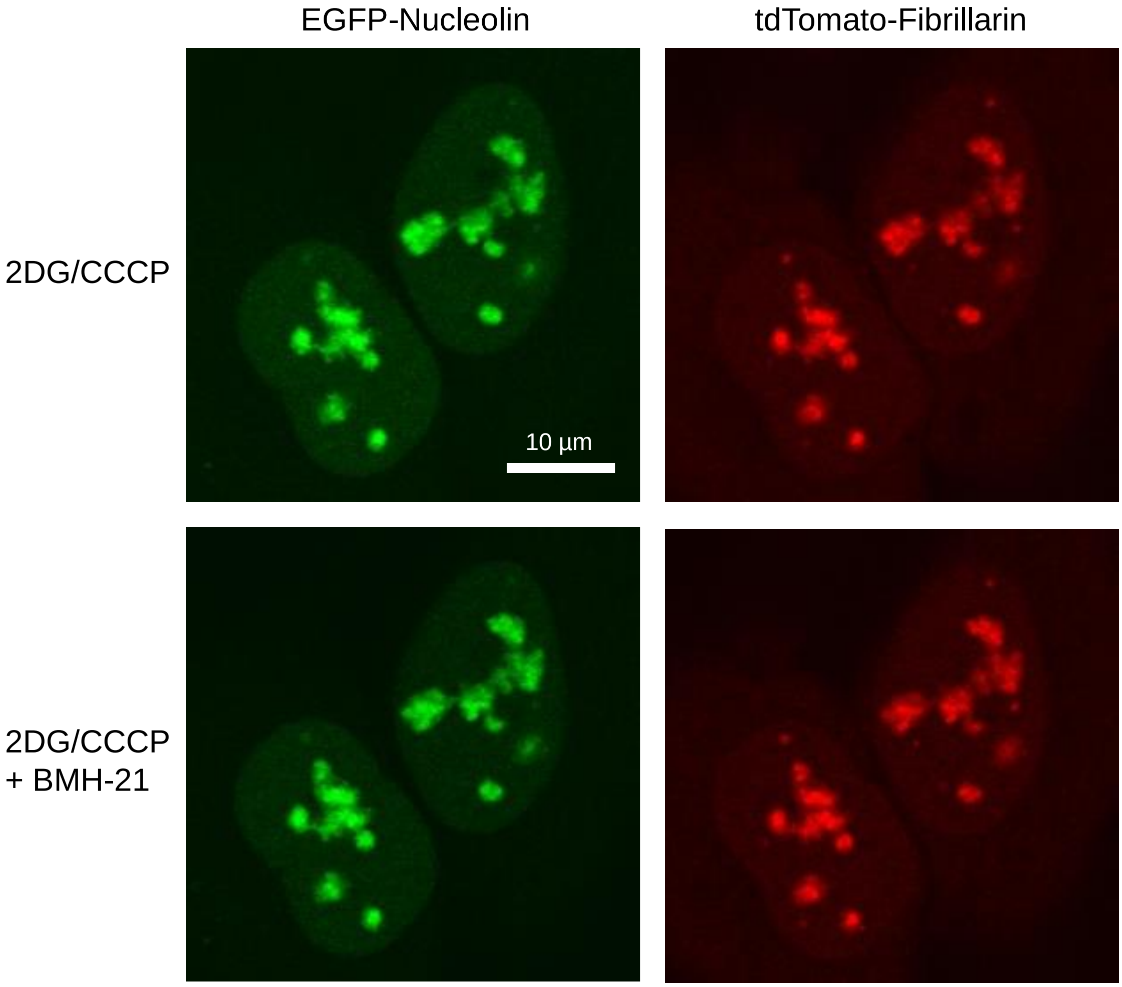

Supplementary Figure 3. BMH-21 does not affect the subnuclear localization of EGFP-Nucleolin and tdTomato-Fibrillarin in 2DG/CCCP-treated cells.
Expression plasmids for EGFP-Nucleolin and tdTomato-fibrillarin were cotransfected into HeLa cells. At 2 days after transfection, cells were treated with 25 mM 2DG and 10 μM CCCP for 20 min. Subsequently, the cells were treated with 1 μM BMH-21 for 5 min in the presence of 2DG and CCCP. Fluorescence images were captured as described in the main text, and representative images are shown.

Supplementary Figure 4

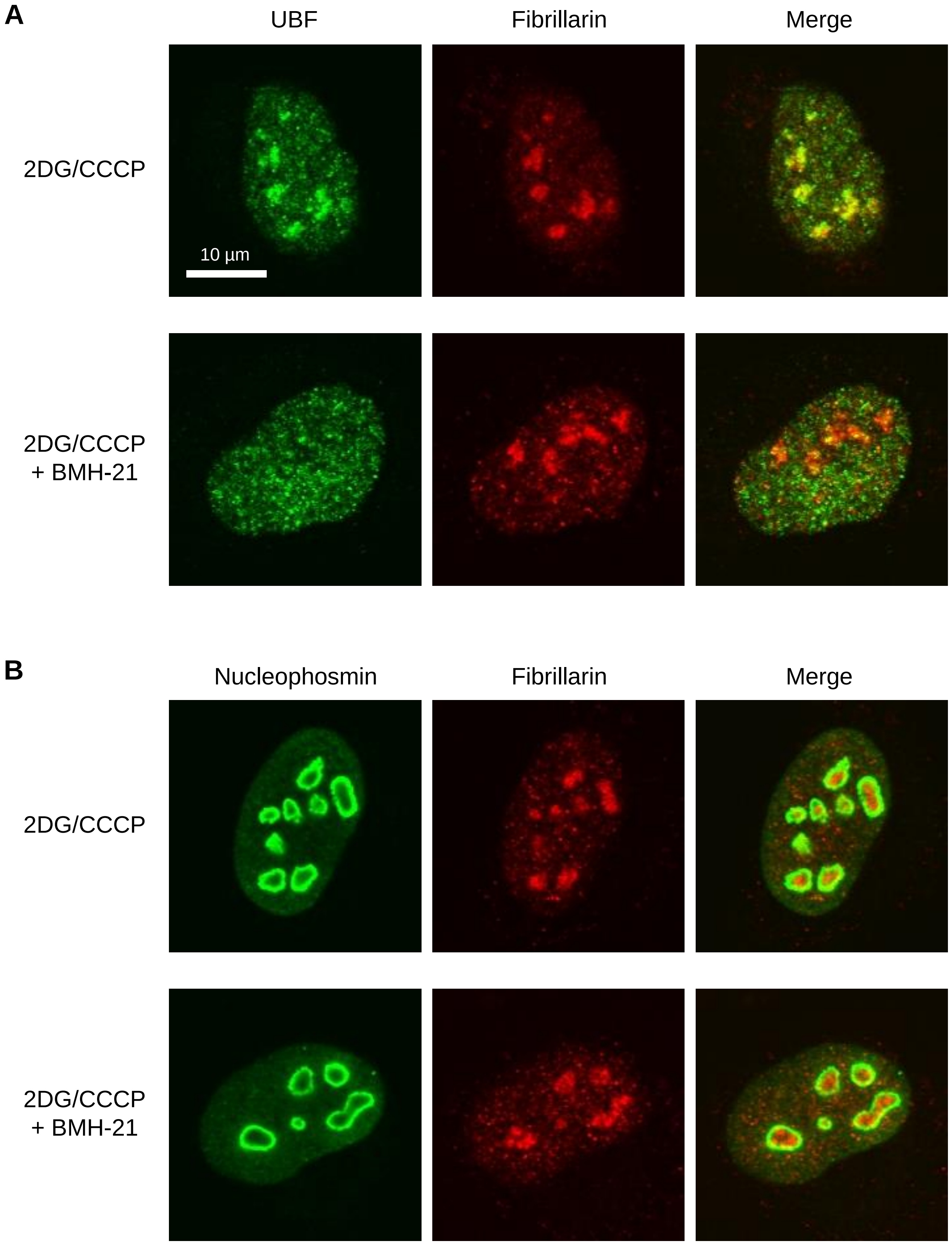

Supplementary Figure 4. Immunofluorescence staining of endogenous nucleolar proteins in 2DG/CCCP- and 2DG/CCCP/BMH-21-treated HeLa cells: Disruption of nucleolar localization of UBF by BMH-21.

HeLa cells were treated with 25 mM 2DG and 10 μM CCCP for 20 min (2DG/CCCP) or treated with 25 mM 2DG and 10 μM CCCP for 20 min and subsequently with 1 μM BMH-21 for 2 min in the presence of 2DG and CCCP (2DG/CCCP + BMH-21). Cells were fixed with 4% paraformaldehyde at 4° C for 15 min. Immunostaining and fluorescence microscopy were performed as described in the main text, and representative images are shown. Following antibodies were used: UBF (sc-13125, Santa Cruz Biotechnology), Fibrillarin (2639S, Cell Signaling Technology), Nucleophosmin (ab10530, Abcam).

A. Immunostaining of UBF and Fibrillarin.

B. Immunostaining of Nucleophosmin and Fibrillarin.

Supplementary Figure 5

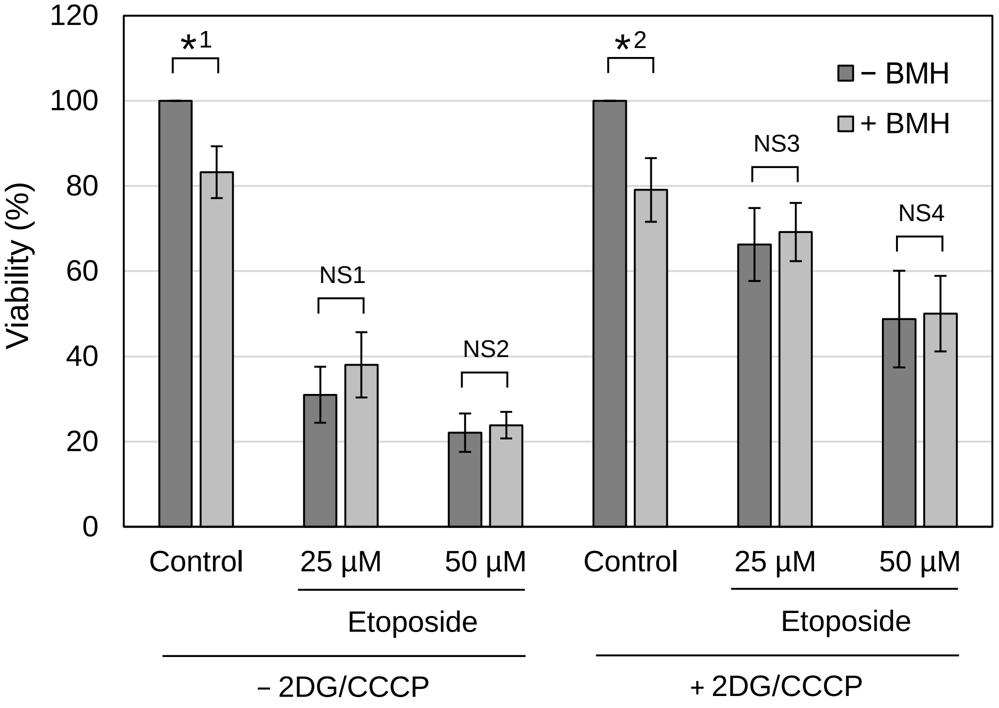

Supplementary Figure 5. Effects of BMH-21 on cell viability.

Cells were treated with a combination of BMH-21 (1 μM), etoposide (25, 50 μM) and 2DG/CCCP (25 mM and 10 μM, respectively) for 30 min, and cell viability was measured as described in the main text. Average values of viability and SD were calculated (n=5). *: statistically significant, NS: Not significant. Statistical values are as follows; *1: p=0.0036, *2: p=0.0033, NS1: p=0.1595, NS2: p=0.5009, NS3: p=0.5707, NS4: p=0.8453.
